# LYP regulates SLP76 and other adaptor proteins in T cells

**DOI:** 10.1186/s40659-024-00536-8

**Published:** 2024-09-28

**Authors:** Virginia Ruiz-Martín, Tamara Marcos, José María de Pereda, Mariano Sánchez-Crespo, Miguel Angel de la Fuente, Yolanda Bayón, Andrés Alonso

**Affiliations:** 1 Unidad de Excelencia Instituto de Biología y Genética Molecular (IBGM), CSIC-Universidad de Valladolid, c/ Sanz y Forés 3, 47003 Valladolid, Spain; 2 Instituto de Biología Molecular y Celular del Cáncer (IBMCC), CSIC-Universidad de Salamanca, Campus Unamuno, 37007 Salamanca, Spain

**Keywords:** Protein tyrosine phosphatase (PTP), LYP (lymphoid phosphatase), T-cell, T-cell receptor (TCR), SLP76, SKAP2, FYB

## Abstract

**Background:**

The LYP tyrosine phosphatase presents a SNP (1858C > T) that increases the risk of developing autoimmune diseases such as type I diabetes and arthritis. It remains unclear how this SNP affects LYP function and promotes the development of these diseases. The scarce information about LYP substrates is in part responsible for the poor understanding of LYP function.

**Results:**

In this study, we identify in T lymphocytes several adaptor proteins as potential substrates targeted by LYP, including FYB, SLP-76, HS-1, Vav, SKAP1 and SKAP2. We also show that LYP co-localizes with SLP76 in microclusters, upon TCR engagement.

**Conclusions:**

These data indicate that LYP may modulate T cell activation by dephosphorylating several adaptor proteins, such as FYB, SLP-76, HS-1, Vav, SKAP1 and SKAP2 upon TCR engagement.

**Supplementary Information:**

The online version contains supplementary material available at 10.1186/s40659-024-00536-8.

## Background

Lymphoid phosphatase (LYP) is a protein tyrosine phosphatase (PTP) expressed in hematopoietic cells: A SNP polymorphism (1858C > T) has been linked to a higher risk of developing several autoimmune diseases, including type I diabetes and systemic lupus erythematosus [1]. Although the evidence supporting this association is compelling, the mechanism underlying the contribution of LYP to autoimmune diseases has been elusive. Similarly, our understanding of the physiological role played by LYP in the immune system remains unclear. The 1854C > T polymorphism changes Arg620 by a Trp in the P1 Pro rich motif (PRM) found at the C-terminus of LYP. C-terminal Src kinase (CSK), a negative regulator of T-cell receptor (TCR) signaling, is the only protein known to bind this motif. Based on this interaction, a model was proposed in which the concerted action of CSK and LYP inactivated LCK in T cells and possibly other SRC family kinases [2]. According to this model, CSK phosphorylates the inactivating Tyr of LCK (Tyr505), while LYP dephosphorylates the activating Tyr of LCK (Tyr394). LYP also interacts through the C-terminal homology (CTH) PRM with another negative regulator of T cell activation, proline-serine-threonine phosphatase interacting protein 1 (PSTPIP1) [3, 4], in this case, the CTH motif binds to an F-BAR domain, which represents a new mode of interaction of PRM with proteins [5]. Mutations in PSTPIP1 cause rare autoinflammatory diseases such as pyogenic sterile arthritis, pyoderma gangrenosum, and acne (PAPA) [6]. LCK, ZAP70, the ζ chain, Cbl, Vav, and SKAP2 have all been proposed to be substrates of LYP [2, 7–9], but information on the regulation of these substrates by LYP is very limited in most cases. Although LYP function has been primarily studied in T cell activation upon TCR engagement, its function has been extended to other receptors in these cells, including the LFA-1 integrin [10], and to B lymphocytes [11], where it regulates BCR induced signaling pathways. Furthermore, LYP has been shown to regulate the innate immune response by enhancing the positive regulation of IFNγ by TLR4 and the activation of NLRP3 in myeloid cells [12, 13].

The purpose of this study was to identify substrates targeted by LYP in T cells in order to gain further insight into the role of this phosphatase in the immune response. To find LYP substrates, we use the sequence of the best peptide dephosphorylated by LYP as query to search protein databases [8]. Using this approach, we identified a group of adaptor proteins critical for TCR signaling, including FYB, SLP76, SKAP1, HS1, and Vav.

## Methods

### Cell lines

HEK293 were maintained at 37 °C in Dulbecco’s modified Eagle’s medium supplemented with 10% fetal bovine serum, 2 mM L-glutamine, 100 U/ml penicillin G, and 100 µg/ml streptomycin. Transient transfection of HEK293 cells was carried out using the calcium phosphate precipitation method [14]. Jurkat T cells were grown in RPMI-1640 medium supplemented with 10% fetal bovine serum, 2 mM L-glutamine, 100 units·mL^−1^ penicillin G, and 100 μg·mL^−1^ streptomycin. Jurkat T cells were transfected by electroporation, as described previously [4]. PBLs were purified by centrifugation at 700 g for 30 min on cushions of Ficoll-Hypaque (GE Healthcare, Little Chalfont, UK) from healthy donor buffy coats obtained from the regional blood bank with approval of its ethics committee. Monocytes/macrophages were removed by adherence to plastic overnight at 37 °C. A PTPN22-deficient Jurkat T cell line (494) was generated with CRISPR/Cas9 technology. A PTPN22 vector was used as donor for homology directed recombination. This vector carries two homology arms and an in-frame STOP codon 12 bp downstream of the ATG start site of the gene and a blasticidin resistance cassette. Clones grown under selection with blasticidin are obtained and those that have incorporated the mutation in homozygosis are selected by PCR and sequencing.

### Antibodies

The hemagglutinin (HA) monoclonal antibody (mAb) was from Covance (Berkely, CA USA). The LCK mouse Ab (3A5), and myc Ab (9E10) were from Santa Cruz Biotechnology Inc. (Santa Cruz, CA). The ABL Ab was from BD Pharmingen (Franklin Lakes, NJ). The phosphotyrosine 4G10 mAb was from Millipore (Billerica, MA). LCK (3A5) from Santa Cruz sc-433 Mab. PSTPIP1 Ab was generated against the whole protein produced in bacteria [4]. Anti-V5 Ig (mouse monoclonal IgG2a) from Invitrogen (Carlsbad, CA, USA). SLP76 pY128 antibody was from Cell Signalling, SLP76 from BD #610736 clone 8/SLP-76, LAT pTyr191 rabbit antibody was from Cell Signalling #3584, LAT antibody Rabbit Mab was from Cell Signalling #45533, and Pyk2 from Cell Signalling #3292. Anti-ERK2 Ig (rabbit polyclonal, C14) from Santa Cruz Biotechnology (CA, USA), and P-ERK1/2 (Thr202/Tyr204) from Cell Signalling #9101. Anti-CD3 Ig (mouse monoclonal IgG1, UCHT1) and anti-CD28 Ig (mouse monoclonal IgG1, CD28.2) from BD Pharmingen (Franklin Lakes, CA, USA); anti-LYP Ig (goat polyclonal, AF3428) from R&D Systems (Minneapolis, MN, USA).

### Plasmids and mutagenesis

Standard molecular biology techniques were used to generate the different plasmids used in this study. All constructions and mutations were verified by nucleotide sequencing. FYB and PSTPIP1 plasmids have been described before [4, 15]. Abl was a gift from Stephen Goff. SKAP-HOM plasmid was a gift from Annegret Reinhold. SKAP55 was amplified in our lab from Jurkat cDNA. SLP76 was a gift from Gary Koretzky, HS1 DNA was from Janis K. Burkhardt and Daniel D. Billadeau, Vav plasmids were kindly provided by Xosé Bustelo, Dok1 and Dok2 were a gift from Jacques A. Nunes, HPK1 construct was from Prof. Dr. Friedemann Kiefer and Arthur Weiss.

### Immunoprecipitation, SDS PAGE and immunoblotting

These procedures were done as reported before [16]. Briefly, cells were lysed in TNE lysis buffer: 20 mM Tris/HCl pH = 7,4, 150 mM NaCl, 5 mM EDTA containing 1% NP-40, 1 mM Na3VO4, 10 µg/ml aprotinin and leupeptin, and 1 mM PMSF, pH 7.5, and clarified by centrifugation at 15,000 rpm for 10 min. The clarified lysates were preabsorbed on protein G-Sepharose and then incubated with Ab and protein G-Sepharose beads for 1 h. Immune complexes were washed three times in TNE buffer and suspended in SDS sample buffer. Proteins resolved by SDS-PAGE were transferred electrophoretically to nitrocellulose membranes, which were immunoblotted with optimal dilutions of specific Abs, followed by the appropriate anti-IgG-peroxidase-conjugate. Blots were developed by the enhanced chemiluminescence technique with Pierce ECL Western Blotting substrate (Thermo Scientific, Rockford IL) according to the manufacturer’s instructions.

### Immunofluorescence and confocal microscopy

Jurkat cells or PBLs were stimulated in 12 mm circular glass coverslips coated overnight at 4 °C with a 10 µg/µl CD3 antibody solution in PBS. After washing the coverslips with 2 ml PBS three times, cells were deposited on the coverslip and incubated at 37 °C for the time of stimulation that was terminated by placing the coverslip on ice. Coverslips were made fresh for each experiment. The cells were then washed in PBS with 0.2% bovine serum albumin (BSA) before being fixed with 4% paraformaldehyde in PBS for 30 min and permeabilized with 0.3% Tritón X-100 for 10 min. Then, cells were incubated in blocking buffer (10% FBS in PBS) for 20 min. Afterwards, the coverslips were incubated with the appropriate primary antibody in PBS with 1% BSA for 1 h, followed by incubation with the corresponding secondary antibody for 1 h, both in PBS with 1% BSA. Cell nuclei were counterstained with 1 μg/ml of the DNA binding dye DAPI added with the secondary antibody. Cells were washed twice with PBS between the different steps. All images were captured with a Leica confocal system TCS SP5X inverted microscope with a HCS Plan Apo CS 63X/1.4 NA oil immersion lens. Leica Application Suite Advanced Fluorescence software was used for the capture, and ImageJ for image presentation. Sections acquired of the stained cells were close to the cell-coverslip interface.

### Purification of LYP

LYP was purified from a stable cell line created in HEK293 cells that express either 3xFLAG-LYP-R620 or 3xFLAG-LYP-W620. Usually, we lysed between 250 and 300 10^6^ cells in 6–8 mL TNE lysis buffer with protease inhibitors, 10% glycerol and 0.5 mM DTT. After clearing the lysate by centrifugation 10 min at 13,200 rpm and 4 ºC, LYP was immunoprecipitated with 200 μL of EZview Red anti-FLAG M2 beads for 1 h at 4 ºC. Beads were then washed three times with NaCl 0.5 M followed by another 3 times with TNE lysis buffer. LYP was eluted from the beads with a 3xFLAG peptide at 0.2 mg/mL in lysis buffer for 20 min at 4 ºC. Elution was done a total of 3 times, collecting a volume of 700–750 µL. Then, protein was concentrated by centrifugation with a filter Amicon Ultra-0.5 mL 30 K (Ultracel-30 K Membrane) up to a final volume of 25–30 μL. Protein was kept at 4 ºC for assays in the following days for a period no longer than a week.

### Phosphatase assays (Protein dephosphorylation assays)

Proteins subjected to be used as substrates for LYP were obtained by immunoprecipitation from HEK293 cells transfected transiently with the plasmids that express them and treated with pervanadate (PV) for 5 min to phosphorylate them. After IP the beads were washed 3 times with TNE lysis buffer and with then another 3 times with phosphatase assay buffer (Sodium acetate 100 mM, Tris 50 mM, Bis–Tris 50 mM, EDTA 5 mM and DTT 5 mM). After the final wash, the SN was removed. Then, 105 μL phosphatase buffer with 2 μL of 3xFLAG-R or W. Tubes were incubated at 37 ºC and aliquots of 24 μL were taken at 0, 5, 15 and 30 min and mixed with 8 μL of 4 × Laemli buffer. Protein phosphorylation was checked by Western Blot with 4G10 specific antibody.

### Luciferase assays

Luciferase activity in Jurkat cells was determined as previously described [4]. Briefly, 20 × 10^6^ Jurkat cells were electroporated with the indicated plasmids, as well as 5 μg of the luciferase gen driven by the IL-2 minimal promoter and 0.5 μg of a Renilla luciferase reporter, which was used for normalization. The following day cells were stimulated with antibodies targeting CD3 and CD28 receptors for 6 h. The cells were then lysed, and the lysates were clarified by centrifugation at 16,000 g for 10 min before being used to measure luciferase activity with the Dual Luciferase system (Promega), according to the manufacturer's recommendations. The statistical significance of the data was determined with Student's t-test, and is indicated in the figures.

## Results

### Identification of substrates of LYP in T cells

Zhang and coworkers found that the best peptide sequence dephosphorylated by LYP was YGEEpYDDLY using inverse Ala scanning (8). To find LYP substrates, we searched the PhosphositePlus database (17) with a degenerate version of Zhang's peptide in which the phospho-Tyr (p-Tyr) is surrounded by acidic amino acids, either D or E, in positions −2 to + 2. Based on the hits obtained, we chose for further investigation proteins expressed in T cells that contain phosphopeptides flanked by at least two acidic residues that are (Fig. S1A).

Prior to investigate the interaction of these proteins with LYP, we determined which substrate-trapping version of this phosphatase might be more adequate for these assays [17]. As a result, we developed LYP mutants in key catalytic amino acids: D195A (DA), C227S (CS), and the double mutant D195A/C227S (DACS). We tested the interaction of these mutants with SKAP2, which contains the closest peptide (Tyr75) to the best LYP substrate [8]. The interaction was tested in transiently transfected HEK293 cells using immunoprecipitation (IP). After PV treatment for 5 min to induce SKAP2 tyrosine phosphorylation, LYP was immunoprecipitated, and the presence of SKAP2 in the precipitates was detected by Western blot (Fig. S1B). In line with previous findings, the substrate trapping mutant that showed the highest interaction with SKAP2 was LYP-DACS [7]. Next, we tested the ability of the DACS mutant to bind putative substrates. To avoid protein interactions with other regions of LYP, we transfected Jurkat cells with a construct that only contains the LYP phosphatase (LP) domain with the double mutation (LP-DACS). PV was applied for 5 min to Jurkat cells, and the LP-DACS peptide was immunoprecipitated. Proteins present in the precipitate were detected by Western Blot with a phospho-Tyr (pTyr) specific antibody. This assay showed that LP-DACS was able to bind to Tyr phosphorylated proteins (Fig. S1C). Based on these results, we used LP-DACS substrate trapping mutant to test the interaction of LYP with the potential substrates found in the database search.

**Fig. 1:**
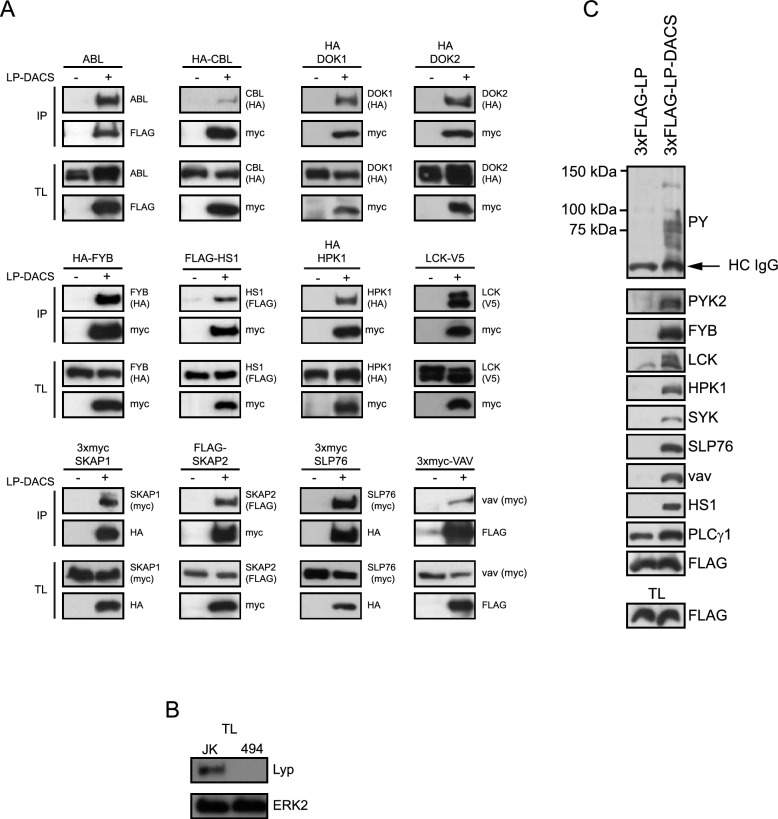
Interaction of the substrate trapping mutant of LYP phosphatase domain, LP-DACS, with several potential substrates. **A** HEK293 cells were transiently transfected with 3xmyc, 3xHA or 3xFLAG-LP-DACS along with plasmids that express potential substrates, as indicated on the top of each panel. After PV treatment for 5 min, cells were lysed and LP-DACS was immunoprecipitated with specific antibodies against myc, HA or FLAG epitopes bound to sepharose beads. Proteins were transferred to nitrocellulose membranes after SDS-PAGE, and the presence of potential substrates in the precipitates was detected by Western Blot. **B** Detection of LYP and ERK2, as loading controls, in Jurkat cells and the LYP-deficient Jurkat-cell line (494). **C** Jurkat cells deficient in LYP (494) were transiently transfected with plasmids that express 3xFLAG-LP, the active phosphatase domain of LYP, and 3xFLAG-LP-DACS. After PV treatment for 5 min, the phosphatase domain of LYP was immunoprecipitated with FLAG antibody bound to Sepharose beads, and proteins present in the precipitates were identified by Western Blot. *HC-IgG* heavy chain IgG, *IP* immunoprecipitates, *TL* total lysates

Following that, we examined the interaction of the potential substrates with LP-DACS in HEK293 cells transiently transfected with plasmids expressing these proteins. After treatment with PV for 5 min, LP-DACS was immunoprecipitated and the presence of the putative substrates detected by Western Blot. In addition to the proteins found from the database search (Fig. S1A), we tested proteins known to participate in signal transduction pathways in immune cells. Altogether, LP-DACS immunoprecipitated the following proteins: ABL, CBL, DOK-1, DOK-2, FYB, HPK1, HS1, LCK, SKAP1, SKAP2, SLP76, and Vav (Fig. 1A). Among the proteins assayed that did not interact with LP-DACS were: CRKL, WASP, CSK, Grb2, ITK, PSTPIP1, p85 subunit of PI3K, LAT, PKA, and the ζ-chain (data not shown and Fig. S2A). To demonstrate the specificity of these assays, we tested the interaction of the substrate trapping mutant LP-DACS with SKAP2, LCK, and PSTPIP1 in the same experiment (Fig. S2A). These results suggest that the proteins detected in the precipitates are likely LYP substrates and warrant further investigation.

To confirm that the interactions detected in HEK293 cells are also found in T cells, we transfected Jurkat cells deficient in the LYP phosphatase (JK 494) with LP-DACS (Fig. 1B). After treatment of Jurkat cells with PV for 5 min, the LP-DACS peptide was immunoprecipitated and the proteins present in the precipitates were detected by Western blot. This way, we found FYB, HPK-1, Vav, SLP76, LCK, HS1, Pyk2, and Syk in LP-DACS precipitates (Fig. 1C). Although these data do not demonstrate a direct association between LYP and these proteins, they indicate that LYP is linked to a number of proteins involved in TCR signaling that share phosphopeptides with similar sequences [18].

### Dephosphorylation of putative substrates by LYP

**Fig. 2:**
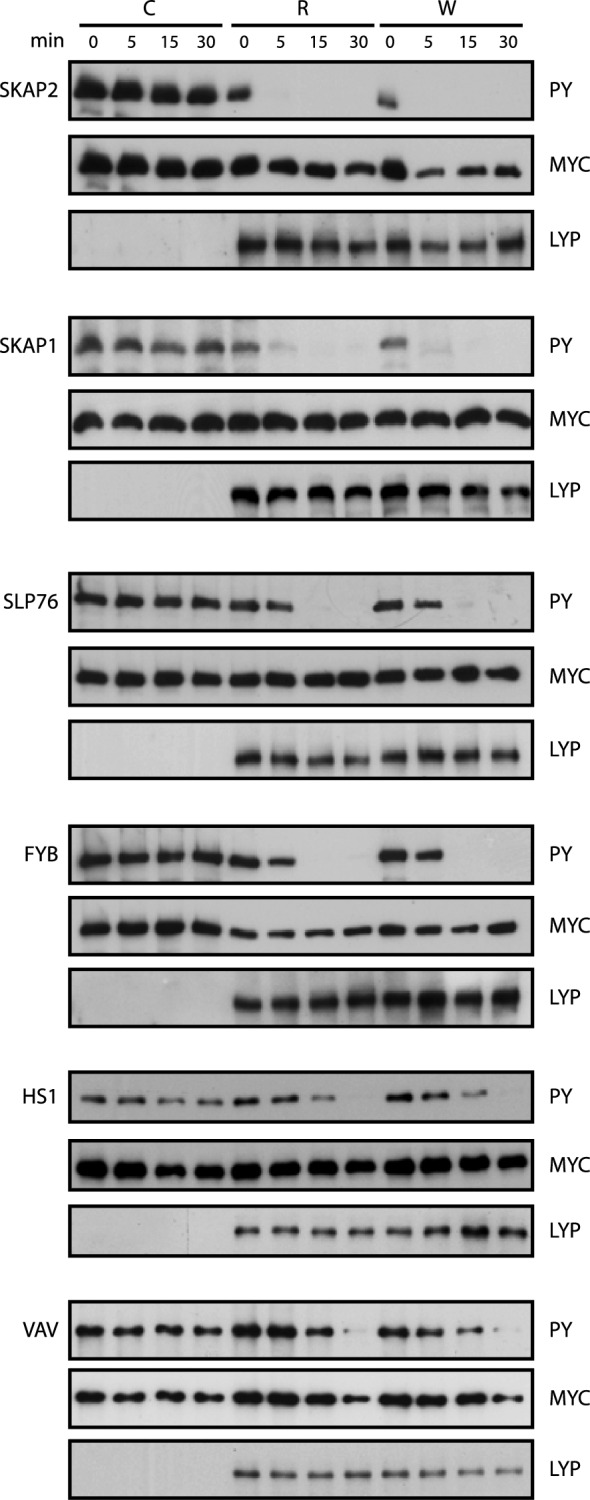
In vitro dephosphorylation assays with LYP-R620 and LYP-W620. Several proteins were tested for dephosphorylation by either LYP-R or LYP-W at the time points indicated on the top of the panel. Phosphatase assays were carried out with a full-length version of LYP and its putative substrates as indicated in Material and Methods. Dephosphorylation was detected by Western blot with p-Tyr (4G10) antibody

Then, we performed in vitro phosphatase assays with full-length LYP to see whether the proteins that interacted with LP-DACS were dephosphorylated by this phosphatase. LYP was purified from a stable cell line established in HEK293 cells by transfection of 3xFLAG-LYP. Substrates were transiently transfected in HEK293, phosphorylated by co-expression with Tyr kinases or PV treatment for 5 min, immunoprecipitated and incubated in phosphatase buffer for 0, 5, 15 and 30 min with either LYP-R620 (LYP-R) or LYP-W620 (LYP-W), the polymorphic variant associated with autoimmune diseases. Dephosphorylation was detected by Western blot with P-Tyr antibody (4G10) (Fig. 2). The fastest dephosphorylation was detected in SKAP2 and SKAP1, followed by FYB and SLP76. HS1 and Vav were also dephosphorylated, although at a slower rate. On the other hand, we did not detect changes in Tyr phosphorylation in HPK-1, LCK, PSTPIP1, and WASP proteins (Fig. S2B).

Given that some proteins dephosphorylated by LYP contain SH3 domains (SKAP2, SKAP1, HS1, Vav and FYB), and that LYP presents several PRMs, we wonder whether LYP could bind to these proteins through their SH3 domain. First, we compared in the same assay the interaction of LP-DACS with these proteins by immunoprecipitation after PV treatment for 5 min (Fig. S3A). In this assay, the proteins with the highest binding were SKAP2 and LCK, while the proteins with the lowest binding were Vav and HS1. As before, PSTPIP1 did not bind to LP-DACS, in keeping with the data obtained in the phosphatase assay. The interaction of these proteins goes in parallel to the phosphatase activity shown previously (Fig. 2). Then, we tested the interaction of active LYP with these proteins to explore whether they could interact through additional regions other than the PTP domain of LYP (Fig. S3B). SKAP1 and HS1 clearly bound to LYP, in addition to CSK and PSTPIP1, two proteins known to bind to LYP PRMs [4, 19], while SKAP2 showed a weaker binding. However, we did not detect in this assay the binding of Vav, SLP76 and FYB, suggesting that the interaction of LYP with these proteins only occurs through the phosphatase domain, and, therefore, LYP and these substrates are put together by adaptor proteins.

### Sites targeted by LYP in putative substrates

**Fig. 3:**
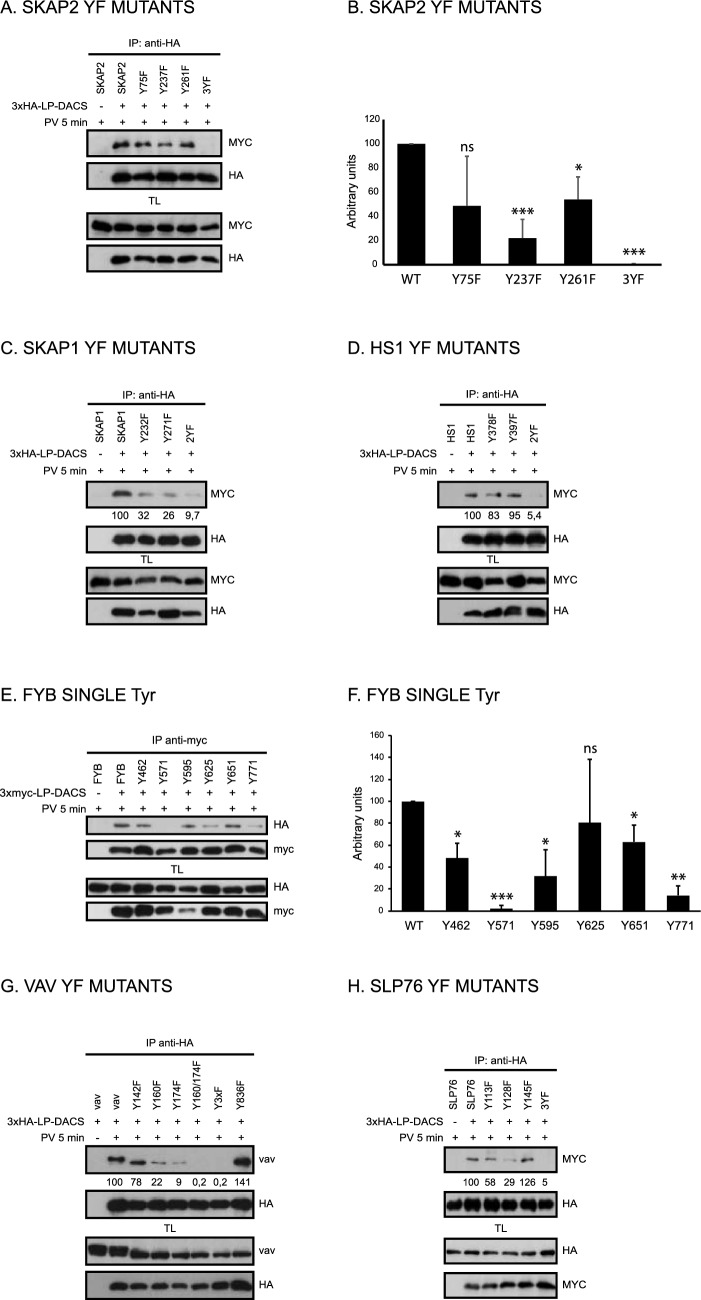
Identification of the Tyr targeted by LYP in several potential substrates. **A** HEK293 cells were transiently transfected with 3xHA-LP-DACS along with expression plasmids of SKAP2 wild type and mutated to Phe in the Tyr indicated. After PV treatment for 5 min, cells were lysed, and LP-DACS was immunoprecipitated with HA Ab bound to Sepharose beads. Proteins were separated by SDS-PAGE and transferred to nitrocellulose membranes. The presence of SKAP2 in the precipitates was detected by WB, as indicated. **B** Graph representing the mean data obtained by densitometry in at least three experiments (n = 4 for single Y to F mutant and n = 3 for 3YF) like the one shown in (**A**). Densitometry values of the myc blot on the top, expressed as percentage of the WT are indicated below. **C** As before, SKAP1 wild type and the mutants in Tyr232 and Tyr271 were assayed for binding to LP-DACS. **D** Interaction of HS1 YF mutants with 3xHA-LP-DACS, as before. **E** Interaction of Vav YF, as indicated, with 3xHA-LP-DACS. **F** Graph representing the mean data obtained by densitometry in three experiments like the one shown in (**E**). Densitometry values of the myc blot on the top, expressed as percentage of the WT are indicated below. **G** Interaction of FYB wild type or mutated in five of the six tyrosines tested, as indicated, with 3xHA-LP-DACS. **H** As before, interaction of SLP76 YF full length mutants with 3xHA-LP-DACS. NS nonsignificant, *P < 0.05,**P < 0.01 and ***P < 0.001 for comparison of proteins mutated in Tyr with the wild type protein

Afterwards, to demonstrate the Tyr targeted by LYP in the proteins here studied, we tested the interaction of LP-DACS with Tyr to Phe mutants in the residues identified in the initial database search. As before, immunoprecipitation after PV treatment for 5 min was used to examine these interactions in transiently transfected HEK293 cells. First, we looked at SKAP2, which contains Y75 (DAEDGEEyDDPFAGP), i.e., the protein sequence most similar to the peptide best dephosphorylated by LYP [8]. In addition to Tyr75, we mutated to Phe Y237 (YDERGELyDDVDHPL) and Y261 (QPIDDEIyEELPEEE), both of which are embedded in an acidic environment. LP-DACS immunoprecipitation of these mutants revealed that Y237F had the lowest binding to LP-DACS, followed by Y261F, and Y75F (Fig. 3A, B). To confirm the Y75 result, which was unexpected, we created double mutants of the three Tyr studied in SKAP2 to test the interaction of LP-DACS with any single Tyr of the three. As previously stated, Y237 had the highest binding, followed by Y261, and finally Y75 (Fig. S4A). Next, we focused on SKAP1, a close protein to SKAP2 that presents Y232 and Y271, which align with SKAP2 Y237 and Y261, but lacks a Tyr equivalent to SKAP2 Y75. Mutation Y232F or Y271F in SKAP1 reduced the interaction with LP-DACS, which was mostly abolished when both Tyr were mutated to Phe (Fig. 3C). We followed our analysis with HS1 Tyr 378 and 397 (Fig. 3D). In HS1, both Tyr are required for the interaction with LP-DACS. In the case of Vav, mutants of several Tyr known to be phosphorylated were used in this assay [20]; however, only Y160 and Y174 were critical for the interaction with LP-DACS (Fig. 3G).

Following that, FYB was examined. This protein contains several phosphorylated Tyr [21]. We chose six of them (Y462, Y571, Y595, Y625, Y651 and Y771) based on their similarity to the peptide best dephosphorylated by LYP [8]. Initially, we tested the interaction of LP-DACS with two fragments of FYB, N-terminal FYB (1–450), and C-terminal FYB (401–783). This assay showed that LP-DACS interacts with the C terminal half of FYB (Fig. S4B) that includes the aforementioned 6 Tyr. To further demonstrate this point, we mutated to Phe the 6 Tyr, and tested its interaction with LP-DACS. Mutation of the 6 Tyr to Phe blocked the interaction between LP-DACS and FYB (Fig. S4C). However, when we mutated one by one any of these Tyr, the interaction did not change (Fig. S4D). Then, to see whether there could be differences in the interaction of each Tyr with LP-DACS, we generated mutants that contained only one of the 6 Tyr studied in FYB. Assays with these mutants showed that the least critical Tyr for the association is Y571 followed by Y771 and Y625, while the other 3 Tyr (595, 651 and 771), which have been shown to bind to the SH2 domain of SLP76 [22], presented a similar binding to LP-DACS (Fig. 3E, F).

SLP76 was the next protein we studied. In this case we mutated the three Tyr in its N-terminal acidic domain, Y113, Y128 and Y145, which are known to be crucial for its function in T cells through the interaction with NCK, Vav, and ITK [23]. A triple mutant of these Tyr (3YF) abrogated the interaction of SLP76 with LP-DACS (Fig. 3H). The mutation that produced the higher reduction in the interaction of SLP76 with LP-DACS was Y128F, followed by Y113F and Y145F. In line with this, the Tyr that generated the higher interaction was Tyr128 followed by Tyr113 and Tyr145 (Fig. S4E). These findings suggest that LYP targets pTyr in several proteins involved in TCR signaling that share a similar acidic amino acid sequence.

### LYP regulates TCR signalling downstream of LCK and ZAP70

**Fig. 4:**
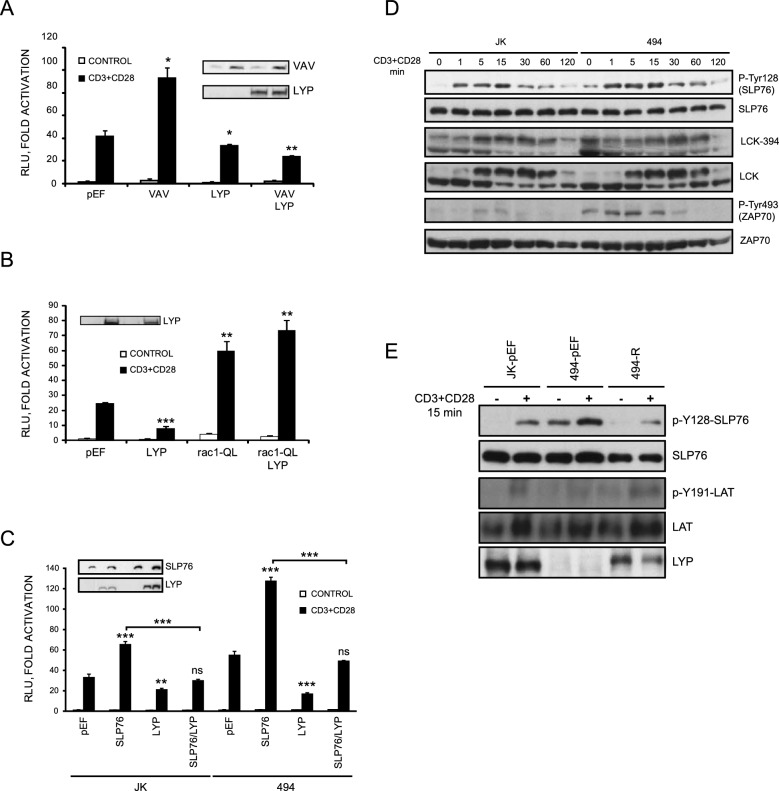
Regulation of TCR signaling by LYP downstream of ZAP70. **A** Activation of a luciferase reporter gene driven by the IL-2 minimal promoter in Jurkat cells transfected with Vav and stimulated with CD3 plus CD28 antibodies for 6 h, as indicated. The insert shows the IB of the Vav and LYP proteins expressed. *P < 0.05 and **P < 0.01 for comparison of cells transfected with different plasmids and cells transfected with empty vector (pEF). **B** Activation of a luciferase reporter gene driven by the IL-2 minimal promoter in Jurkat cells transfected with rac-QL, a dominant active mutant of rac, and LYP and and stimulated with CD3 plus CD28 antibodies for 6 h. The insert shows the IB of LYP. *P < 0.05 and **P < 0.01 for comparison of cells transfected with different plasmids and cells transfected with empty vector (pEF) **C** Activation of a luciferase reporter gene driven by the IL-2 minimal promoter in wild type and 494 Jurkat cells transfected with LYP and SLP76 as indicated. The cells were left untreated or stimulated with CD3/CD28 antibodies for for 6 h. The insert shows the IB of the SLP76 and LYP proteins expressed. *P < 0.05 **P < 0.01 and ***P < 0.001 for comparison of cells transfected with different plasmids and cells transfected with empty vector (pEF). **D** Wild type and 494 JK cells were stimulated with CD3/CD28 antibodies during the indicated periods of time. Phosphorylation of Y128 of SLP76 in each condition was measured in cell lysates by IB. Similarly, phosphorylation of LCK in Y394 and Y493 are shown. E, Wild type Jurkat cells and cells deficient in LYP (JK 494) transfected with LYP were stimulated with CD3/CD28 antibodies for 15 min and the phosphorylation of Y128 of SLP76, and Y191 of LAT were detected in each condition by IB

To determine whether LYP regulates substrates downstream of LCK and ZAP70, like the proteins studied in this work, we examined T cell activation in cells overexpressing Vav, which is recruited by SLP76 [23]. Jurkat cells were transfected with a plasmid expressing Vav as well as a reporter plasmid expressing luciferase driven by the IL-2 promoter [4]. Indeed, LYP expression blocked the activation caused by Vav (Fig. 4A). Then, we tested whether LYP could prevent the activation induced by racQL, a dominant active mutant of the small GTPase rac that is activated by Vav in T cells [24]. In this case, LYP did not prevent luciferase induction (Fig. 4B). Next, we tested whether LYP could affect T-cell activation via SLP76. Transfection of SLP76 increased luciferase induction in response to CD3 plus CD28 stimulation, whereas LYP transfection reduced IL-2 activation induced by SLP76 in stimulated JK cells (Fig. 4C). When these plasmids were transfected in LYP-deficient Jurkat cells (494), SLP76 induced a higher expression of luciferase in comparison with wild type cells. Co-expression of LYP in these cells reduced luciferase induction to the control levels, indicating that the increase in T cell activation in these cells is due to the absence of LYP. These assays collectively revealed that LYP also regulates proteins downstream of LCK and Zap70.

Then, we wanted to test whether LYP deficiency increased SLP76 phosphorylation in cells, using the LYP-deficient Jurkat cell line (494). For this purpose, celIs were stimulated with CD3 and CD28 Abs, and the phosphorylation of Y128 was analysed by Western blot. In these experiments, LYP deficiency increased Y128 phosphorylation in SLP76 (Fig. 4D). In line with previous findings, we found increased phosphorylation in Tyr493 of ZAP70 [25]. Nevertheless, in the case of LCK Tyr394, we found an increase of LCK phosphorylation in resting cells, though the effect upon CD3 stimulation is less clear. We reintroduced LYP phosphatase into 494 cells to confirm that the increase in phosphorylation is LYP specific. In these cells, LYP expression reduced phosphorylation of this protein close to Jurkat parental cells (Fig. 4E). However, when we detected the phosphorylation of Y191 in LAT, we did not see an increase in its phosphorylation in LYP deficient cells. These findings support that LYP targets SLP76 in T cells.

To investigate how LYP can regulate the interaction of SLP76 with its ligands NCK, vav, and ITK via the C-terminal tyrosines 113, 128 and 145, we transfected HEK293 cells and tested by immunoprecipitation if LP-DACS could block this interaction (Fig. S5A). In this assay, we found that LP-DACS inhibited the interaction of NCK, which is primarily mediated by Tyr128, but did not affect the interaction with vav, mediated by Tyr113, and diminished the association with Itk via Tyr 145 [23], which is consistent with previous data that showed that Tyr128 is the tyrosine mainly targeted by LYP in SLP76 (Fig. 3F and Fig. S4E). Furthermore, in a different assay, expression of LYP prevented NCK-SLP76 interaction (Fig. S5B). We further noticed that LYP expression could inhibit the interaction between SLP76 and FYB, but this effect was not seen when an inactive version of LYP (3xFLAG-R or W-DACS) was used in this assay (Fig. S5C). In summary, these assays show that LYP alters the interaction of SLP76 and FYB with other proteins involved in T cell activation.

### LYP regulates the formation of microclusters in T cells

**Fig. 5:**
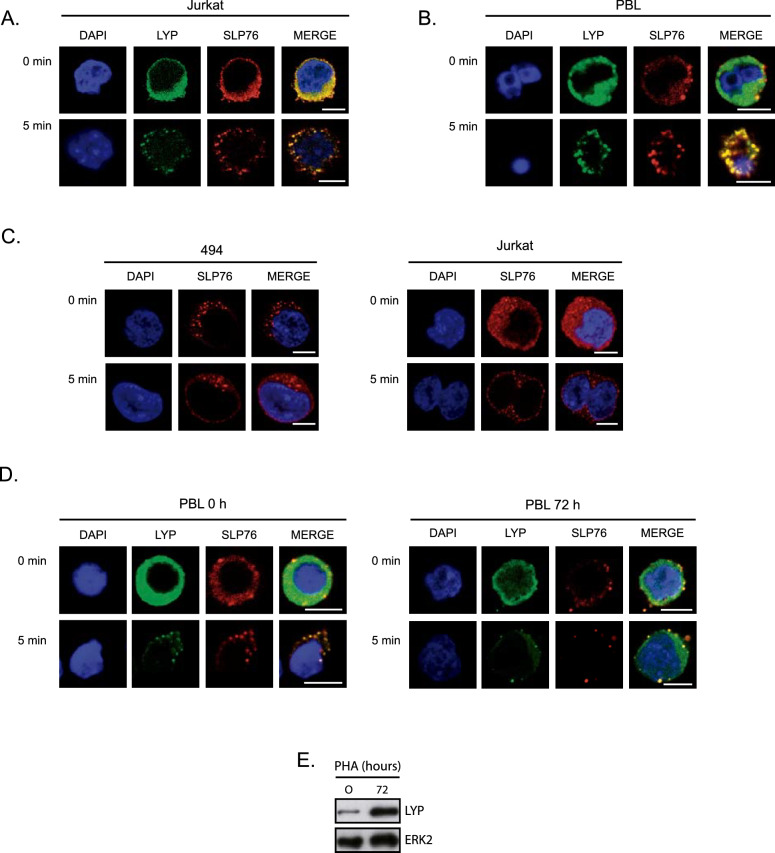
LYP co-localizes with SLP76 in T cell microclusters. Jurkat cells (**A**) or PBL (**B**) were plated on coverslips covered with antibody for CD3 for the indicated periods of time. Then, cells were fixed and stained with specific antibodies for SLP76 and LYP, as indicated. Images were taken with a confocal microscope and representative images are shown. C, Wild type and 494 Jurkat cell lines were plated on coverslips covered with CD3 antibody for the indicated periods of time. Then, cells were fixed and stained with specific antibodies for SLP76. D, PBL cells were left untreated or stimulated with PHA for 72 h to induce the expression of LYP. As before, they were plated on stimulatory coverslips and processed to detect LYP and SLP76 by immunofluorescence with a confocal microscope. E, LYP expression in PLB treated with PHA. Scale bar represents 5 μm

SLP76 is found in microclusters, which are protein complexes organized in T cells to transduce signals upon TCR engagement [22]. Because LYP is a potential negative regulator of SLP76, we studied the association of LYP with SLP76 in these complexes. To this end, we activated Jurkat T cells by placing them on CD3 antibody coated coverslips for different periods of time. Cells were then processed for immunofluorescence to detect the localization of LYP and SLP76. Microclusters began to form in these assays after 3 min of stimulation and were present in all cells after 5 min (data not shown). We found that LYP colocalized with SLP76 in microclusters (Fig. 5A). Then, we investigated whether LYP localization occurred in PBL microclusters and confirmed the results obtained in Jurkat cells (Fig. 5B). These findings suggest that LYP and SLP76 co-localize in microclusters. Next, we wondered whether LYP could regulate the assembly of these complexes upon T cell activation. We use the 494 JK cell line to investigate how LYP deficiency affects microcluster formation. When we stimulated these cells with CD3 antibody coated coverslips, we found that microclusters were present even before proper activation by CD3 antibodies and there was no increase in microcluster formation at 5 min (Fig. 5C). In comparison to 494 cells, there was no aggregation of SLP76 in microclusters in resting JK cells, which was not detected until later (5 min) (Fig. 5C). Then, we explored the role of LYP in microcluster formation in PBL. LYP levels were low in resting PBLs but significantly increased after T-cell activation (Fig. 5D). Thus, in resting PBLs, with low amounts of LYP, there were more microclusters than in PBLs stimulated with PHA for 72 h, which expressed higher amounts of LYP (Fig. 5D). Overall, these findings suggest that LYP associates with SLP76 in T cell microclusters, and regulates their formation, most likely by targeting key Tyr involved in protein interaction.

## Discussion

This study shows that LYP dephosphorylates a number of adaptor proteins involved in TCR signaling, including SLP76, FYB, HS1, Vav, SKAP1 and SKAP2. Tyrosines targeted by LYP in these proteins are surrounded by acidic amino acids. LYP also co-localizes with SLP76 in microclusters when cells are activated via the TCR. LYP deficiency increases SLP76 tyrosine phosphorylation and microcluster formation in T cells.

A LOGO was created using the sequence of the phospho-peptides discovered in the proteins that bind to LP-DACS: SKAP1, SKAP2, SLP76, FYB, Vav, and HS1. (Fig. S6). These peptides exclusively present Asp or Glu in the + 1 position. Although these amino acids are also common between -7 and + 7, they are most noticeable in positions −2 and −3. In + 3 position, hydrophobic amino acids (P, L, V, I) are preferred. Similar results were obtained when an LP-DA trapping mutant was used in phosphopeptide chip arrays [26]. LYP contains an electropositive surface surrounding the catalytic pocket that would explain why acidic residues are preferred [27, 28]. LYP, like PTP1B, has a secondary substrate-binding pocket where the D/E in + 1 position contacts with K32 [8, 29]. Interestingly, the sequences of the peptides targeted by LYP are similar to the sequences phosphorylated by the Tyr kinases LCK and ZAP70: an hydrophobic residue in + 3 position and acidic residues close to the pTyr [21]. Therefore, LYP, dephosphorylates not only LCK and ZAP70 [25], but also their substrates.

Tyr75 in SKAP2 lies in the protein sequence that is most similar to the optimal peptide dephosphorylated by LYP, with the four amino acids closest to the pTyr being Asp or Glu [8]; although in our assays, Tyr75 shows a weaker binding than Tyr237 and Tyr261, with the main difference being the presence of a hydrophobic amino acid in -1 (L or I). SKAP2 tyrosines 237 and 261 align with SKAP1 tyrosines 232 and 271. In contrast, SKAP1 lacks a Tyr similar to Tyr75 of SKAP2. Another PEST phosphatase, PTP-PEST, has been shown to dephosphorylate Tyr261 and Tyr298 in SKAP2 [30].

So far, LCK and ZAP70 have been the most well studied LYP substrates [25]. Both LCK (Tyr394) and ZAP70 (Tyr493) activating tyrosines are dephosphorylated by LYP. Although LYP dephosphorylates LCK in vitro [7], LCK Y394 peptide is a weaker substrate than the SKAP2 Tyr75 peptide [8]. Indeed, the sequence surrounding Y394 in LCK (RLIEDNEytAREGAK) differs from both the optimal sequence dephosphorylated by LYP [8] and the consensus sequence found in this study. LCK presents an R in position + 3, which is restricted to hydrophobic amino acids in the proteins here studied; and a Thr in + 1 instead of an acidic residue. Nonetheless, LCK Thr395 can be phosphorylated [21], allowing it to bind to the secondary binding pocket of LYP [27] and facilitating its interaction to dephosphorylate Tyr394. On ZAP70, Tyr493 is followed by an Arg in + 3, and a Thr in + 1, Thr494, which has also been found to be phosphorylated [21]. Thus, phosphorylation of a threonine in + 1 in LCK and Zap70, could regulate the interaction with LYP and dephosphorylation of nearby tyrosines by this phosphatase. Other proteins proposed as LYP substrates include CD3ε, CD3ζ-chain, the ATPase VCP/p97, Bcr-Abl, C-Cbl and NLRP3 [25]. LYP dephosphorylates NLRP3 in Tyr861 (SHSLTRLyVGENALG) in a noticeably less acidic sequence than the peptides identified in this and previous studies [8, 13]. Despite the fact that LCK is the best studied LYP substrate, it appears that LYP dephosphorylation of LCK does not explain the contribution of LYP to autoimmune disorders. Other phosphatases in T cells regulate LCK, including CD45 (PTPRC), which can dephosphorylate both the activating Y394 and the inhibitory Y505 [31]. Furthermore, LCK activity on T cells does not appear to be entirely dependent on Y394 phosphorylation because it is already phosphorylated in resting cells [32].

The proteins that interact with LP-DACS and are dephosphorylated by LYP (SLP76, FYB, SKAP1, SKAP2, Vav and HS1) participate in the “signal diversification and regulation module” that is part of the T cell signalosome [33]. Once the TCR is engaged, most of these proteins organize into signaling complexes called microclusters, which are nucleated around several adaptor proteins [18]. LAT, a critical adaptor in antigen-induced T cell activation, is phosphorylated by Zap70 and binds to the Grb2-related adapter downstream of Shc (GADS), which is stably associated with SLP76 [34]. Phosphorylation of SLP76 in the N-terminal tyrosines 113, 128 and 145 mediates the interaction of this protein with NCK, Vav and ITK [35]. Additionally, the SH2 domain of SLP76 interacts mainly with Tyr 595, 651 and 771 in FYB/ADAP [22, 36]. NCK, through its SH2 domain, also binds to FYB when is phosphorylated (37). The Pro-rich domain of FYB is constitutively associated with the SH3 domain of SKAP1 and SKAP2 [37]. HS1 is also involved in antigen-induced T cell activation. Its phosphorylation allows binding to various proteins that regulate the actin cytoskeleton via the activation of Vav1 and actin-related proteins-2/3 (Arp2/3) complex [38]. Interestingly, mutations in HS1 have been found in patients with lupus and acute myelocytic leukemia (AML) [38, 39]. Thus, interactions of these proteins, which are critical for T cell activation and are dependent on tyrosine phosphorylation, seem to be regulated by LYP, which rather than targeting a particular protein, dephosphorylates several adaptor proteins that are part of the microclusters.

In this work, we show that LYP can dephosphorylate directly SLP76 in the C-terminal tyrosine 128. Upon TCR engagement, SLP76 is phosphorylated by LCK and ZAP70 [40, 41], and associates in complexes called microclusters, in which some of the proteins here studied as LYP substrates, for instance, Vav1 and FYB, also participate [22, 42]. Previously, it was shown that treatment of T cells with a LYP inhibitor increased SLP76 phosphorylation in Y128 [43], but direct dephosphorylation of this protein by LYP was not studied. Moreover, we also describe for the first time that LYP is localized in microclusters and regulates them. Thus, cells deficient in LYP present microclusters in the resting state, indicating that LYP impairs formation of these complexes and avoids the activation of T cells in the absence of stimuli. The action of LYP on adaptor proteins that organize signaling complexes assembled after TCR stimulation may be critical for the immune response in T cells.

## Conclusions

The data presented in this study support LYP regulation of T cell activation through dephosphorylation of TCR signaling proteins such as SKAP1, SKAP2, SLP76, FYB, Vav, and HS1. As a result, our study advances in the knowledge of the substrates of the phosphatase LYP, and provides clues to define the specificity of this phosphatase, which may also aid in characterizing the specificity of other phosphatases. Since those proteins are expressed in other cells of the immune system, LYP action on them may help to regulate additional functions mediated by these proteins, such as phagocytosis or antibody production.

## Supplementary Information

Supplementary material 1.

## Data Availability

All data generated or analyzed during this study are included in this published article and its supplementary information files.

## Abbreviations

Ab: Antibody
CTH: C-terminal homology domain
CSK: C-terminal Src kinase
IB: Immunoblot
IP: Immunoprecipitation
LCK: Lymphocyte cell-specific protein-tyrosine kinase
LYP: Lymphoid phosphatase
PBL: Peripheral blood lymphocytes
PRM: Proline rich motif
PTP: Protein tyrosine phosphatase
PV: Pervanadate
SFKs: Src family kinases
TCR: T-cell receptor
TL: Total lysate
