# Supplementary material for: LYP regulates SLP76 and other adaptor proteins in T cells
Source: Biol Res. 2024 Sep 28;57:69. doi: 10.1186/s40659-024-00536-8 (PMC11438317; doi:10.1186/s40659-024-00536-8)
Supplement: Supplementary file 1 — Supplementary material 1. [file 40659_2024_536_MOESM1_ESM.pdf]

Supplementary Material

LYP regulates SLP76 and other adaptor proteins in T cells.

Virginia Ruiz-Martín[1], Tamara Marcos[1], José M. de Pereda[2], Mariano Sánchez Crespo[1], Miguel Angel de la Fuente[1], Yolanda Bayón[1*] and Andrés Alonso[1*]

[1]Unidad de Excelencia Instituto de Biología y Genética Molecular (IBGM), CSIC-Universidad de Valladolid, c/ Sanz y Forés 3, 47003 Valladolid, Spain.
[2]Instituto de Biología Molecular y Celular del Cáncer (IBMCC), CSIC-Universidad de Salamanca, Campus Unamuno, 37007, Salamanca, Spain

[*]To whom correspondence should be addressed: Andrés Alonso, Instituto de Biología y Genética Molecular (IBGM), CSIC-Universidad de Valladolid, c/ Sanz y Forés 3, 47003 Valladolid, Spain Tel: +34-983-184839 Fax: +34-983-184800; E-mail: andres.alonso.garcia@uva.es; or Yolanda Bayón, Instituto de Biología y Genética Molecular (IBGM), CSIC-Universidad de Valladolid, c/ Sanz y Forés 3, 47003 Valladolid, Spain Tel: +34-983-184839 Fax: +34-983-184800; E-mail: ybayon@ibgm.uva.es

A

BEST LYP SUBSTRATE	YGEEyDDLY
ABL Y257-p	GGQyGEVyEGVWKKy
CBL Y141-p	kEGKERMyEENSQPR
CBL Y368-p	IKVTQEQyELyCEMG
CBL Y371-p	TQEQyELyCEMGSTF
CBL Y455-p	EGAPsPNyDDDDER
CBL Y774-p	sENEDDGyDVPKPPV
CD3Z Y83-p	NLGRREEyDVLDkRR
CD3Z Y123-p	kDkMAEAysEIGMkG
CD3Z Y153-p	stAtKDtyDALHMQA
DOK1 Y362-p	DPKEDPIyDEPEGLA
DOK1 Y409-p	yNPAtDDyAVPPPRS
DOK2 Y345-p	PPRPDHIyDEPEGVA
FYB Y462-p	QDSEGETyEDIEASK
FYB Y571-p	TTAVEIDyDsLKLKK
FYB Y595-p	IEDDQEVyDDVAEQD
FYB Y625-p	PPPDDDIyDGIEEED
FYB Y651-p	LDMGDEVyDDVDTSD
FYB Y771-p	ADNDGEIyDDIADGC
HPK1 Y381-p	SESSDDDyDDVDIPT
HS1 Y378-p	EPEPENDyEDVEEMD
HS1 Y397-p	EDEPEGDyEEVLEPE
ITK Y198-p	ALRRNEEyCLLDSSE
LAT Y156-p	ADEDEDDyHNPGyLV
LAT Y200-p	SMESIDDyVNVPEsG
LAT Y255-p	EEEGAPDyENLQELN
SKAP2 Y75-p	DAEDGEEyDDPFAGP
SKAP2 Y237-p	YDERGELyDDVDHPL
SKAP2 Y261-p	QPIDDEIyEELPEEE
SKAP1 Y232-p	EEEKEETyDDIDGFD
SKAP1 Y271-p	EKEEEDIyEVLPDEE
SLP76 Y113-p	SSFEEDDyESPNDDQ
SLP76 Y128-p	DGEDDGDyESPNEEE
SLP76 Y145-p	PVEDDADyEPPPSND
VAV1 Y160-p	VEEDEDLyDCVENEE
VAV1 Y174-p	EAEGDEIyEDLMRSE
VAV1 Y841-p	ANYVEEDYSEYC
PIK3R1 Y467-p	SREyDRLyEEytRTS
PIK3R1 Y470-p	yDRLyEEytRTSQEI
PRKACA Y331-p	DTSNFDDyEEEEIRV

B

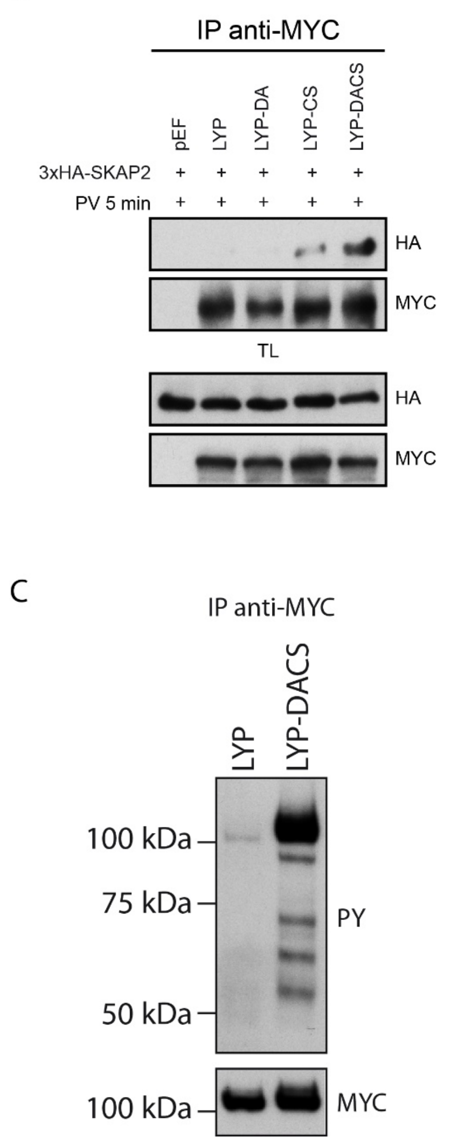

Supplementary Figure 1. New putative substrates of the LYP tyrosine phosphatase. A, Peptides with at least two acidic amino acids shown in red, Asp or Glu, surrounding the phosphor-Tyr in the motifs found after searching the PhosphositePlus database with the peptide identified by Zhang's lab as query (1), included at the top. B, Interaction of different substrate trapping mutants of LYP, D195A (DA), C227S (CS) and the double mutant D195A/C227S, (DACS), with SKAP2 by immunoprecipitation of HEK293 transiently transfected with the plasmids that express these proteins after pervanadate (PV) treatment for 5 min, as indicated in the panel. The presence of SKAP2 in the precipitates was detected by Western Blot. C, Assay to test the capacity of the substrate trapping mutant of LYP, LYP-DACS, to bind Tyr phosphorylated proteins. Jurkat T-cell leukemia cells were transfected with pEF3xFLAG-LP-DACS construct and after PV treatment for 5 min lysates were immunoprecipitated with antibody (Ab) for the FLAG epitope. Proteins bound were detected by Western Blot (WB) with a Phospho-Tyr (4G10) specific Ab.

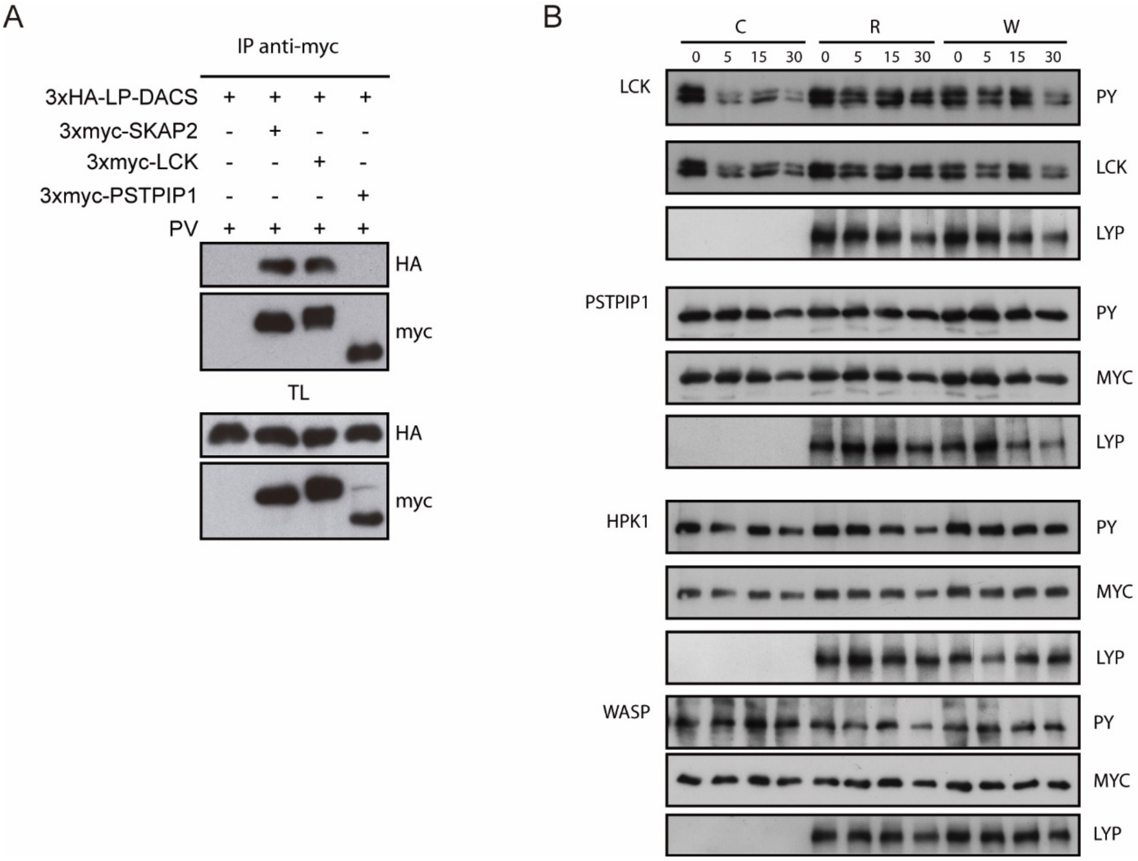

Supplementary Figure 2. Specificity of LYP phosphatase. A, HEK293 cells were transiently transfected with 3xHA-LP-DACS along with expression plasmids for several putative substrates of LYP: SKAP2, LCK and PSTPIP1, expressed with a 3xmyc tag, as indicated. After PV treatment for 5 min, cells were lysed and LP-DACS was immunoprecipitated with myc Ab bound to Sepharose beads. After SDS-PAGE, proteins were transferred to nitrocellulose membranes and detected by WB with HA and myc antibodies. B, *In vitro* dephosphorylation assays with LYP-R620 and LYP-W620 for proteins LCK, PSTPIP1, HPK1 and WASP. Phosphatase assays were carried out with a full-length version of LYP as indicated in Material and Methods. Dephosphorylation was detected by Western blot with p-Tyr (4G10) antibody after different times of incubation, as indicated on top of the panel.

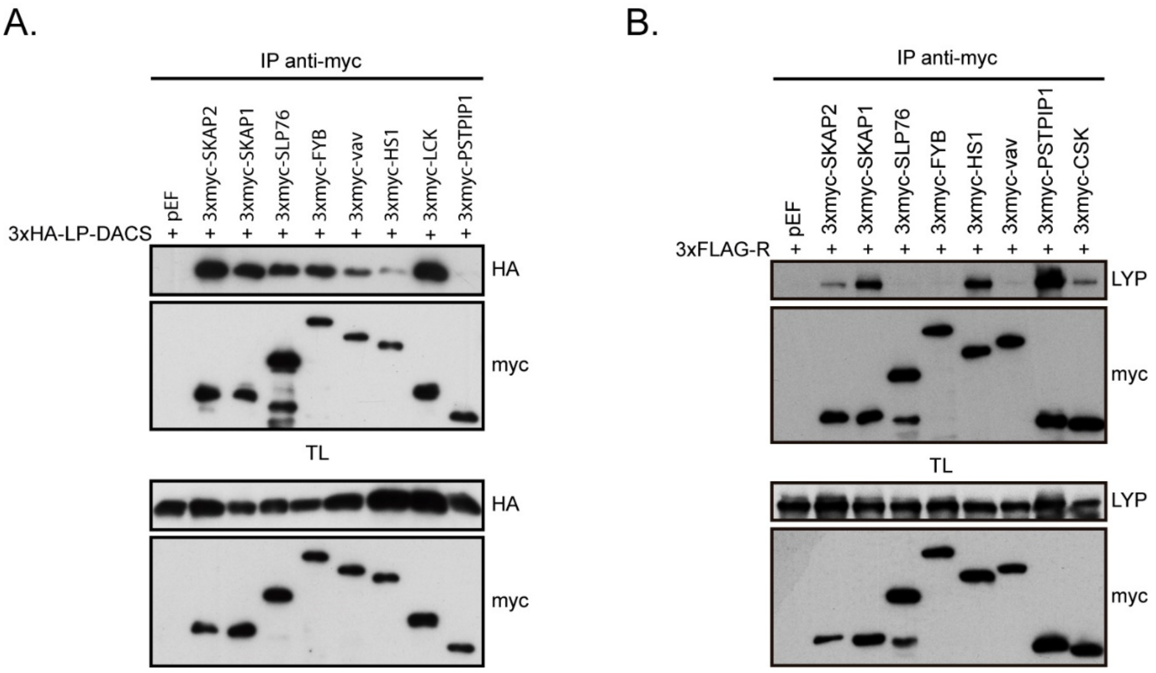

Supplementary Figure 3. Interaction of LYP with its substrates. A, HEK293 cells were transiently transfected with 3xHA-LP-DACS along with expression plasmids for several putative substrates of LYP expressed with a 3xmyc tag, as indicated. After PV treatment for 5 min, cells were lysed and LP-DACS was immunoprecipitated with myc Ab bound to Sepharose beads. After SDS-PAGE, proteins were transferred to nitrocellulose membranes. The presence of the potential substrates in the precipitates was detected by WB with HA antibody. B, as in A, but in this case, LYP wild type in a full-length version was transfected with the putative substrates of LYP tested in A.

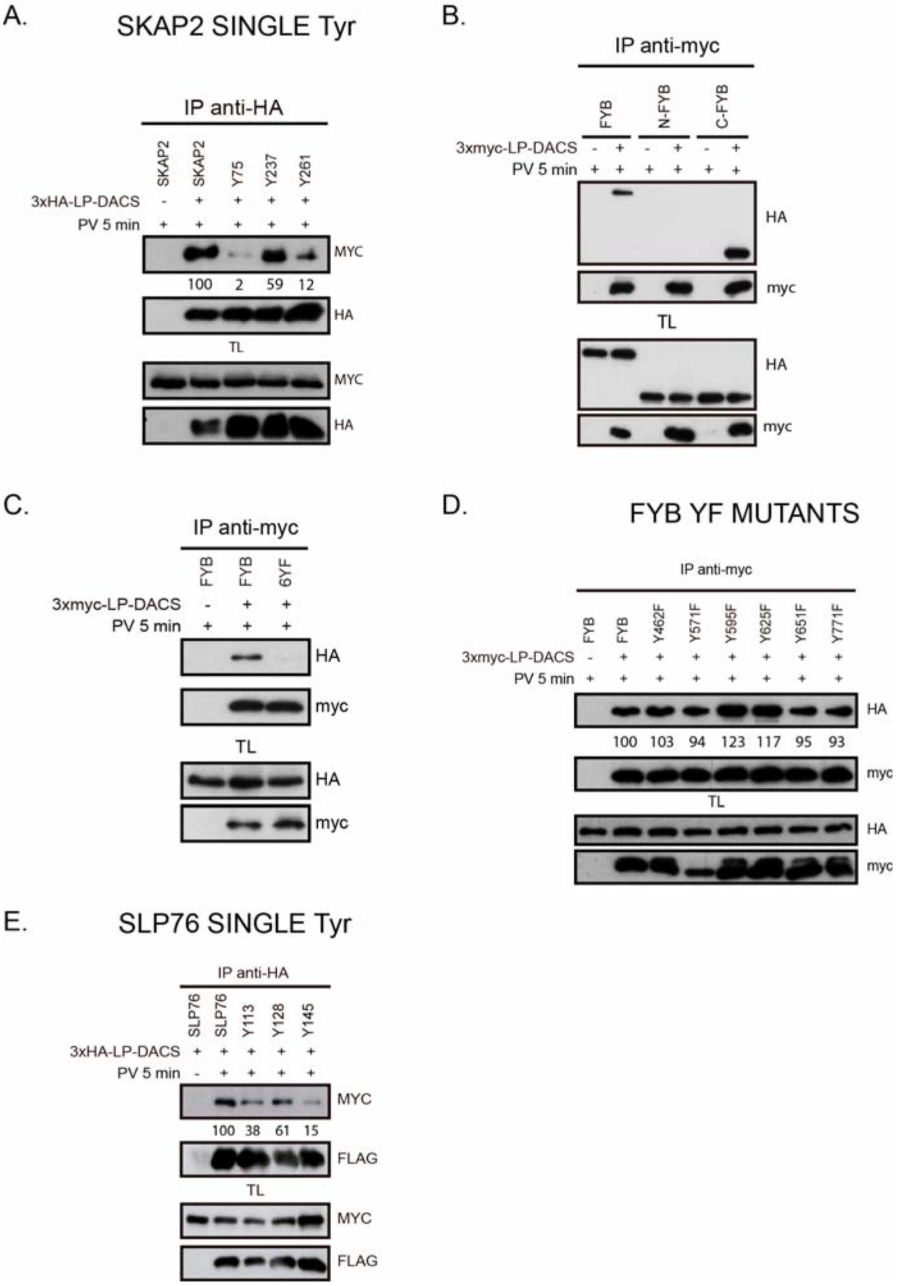

Supplementary Figure 4. Tyr targeted by LYP in potential substrates. A, HEK293 cells were transiently transfected with 3xHA-LP-DACS along with expression plasmids of SKAP2 wild type and Tyr to Phe mutants to leave only the Tyr indicated in the panel. After PV treatment for 5 min, cells were lysed, and LP-DACS was immunoprecipitated with HA Ab bound to sepharose beads. Proteins were separated by SDS-PAGE, and transferred to nitrocellulose membranes. The presence of SKAP2 in the precipitates was detected by WB, as indicated. Densitometry values were obtained with Image J for the interaction and values were expressed as percentage of the wild type protein (WT) and indicated below the blot. B, As before, FYB full length as well as the n and C.-terminal halves were assayed for binding to LP-DACS. C, Interaction of FYB wild type and mutated in the six tyrosines studied in this protein to Phe with 3xHA-LP-DACS, as before. D, Interaction of FYB with only one out of the six Tyr studied to show the interaction with 3xHA-LP-DACS, as the previous assays. Densitometry values for the interaction as in A are indicated below the top blot. E, Interaction of SLP76 wild type and mutants with one Tyr out of three, as before. Densitometry values for interaction of the Tyr to Phe mutants are indicated in the panel.

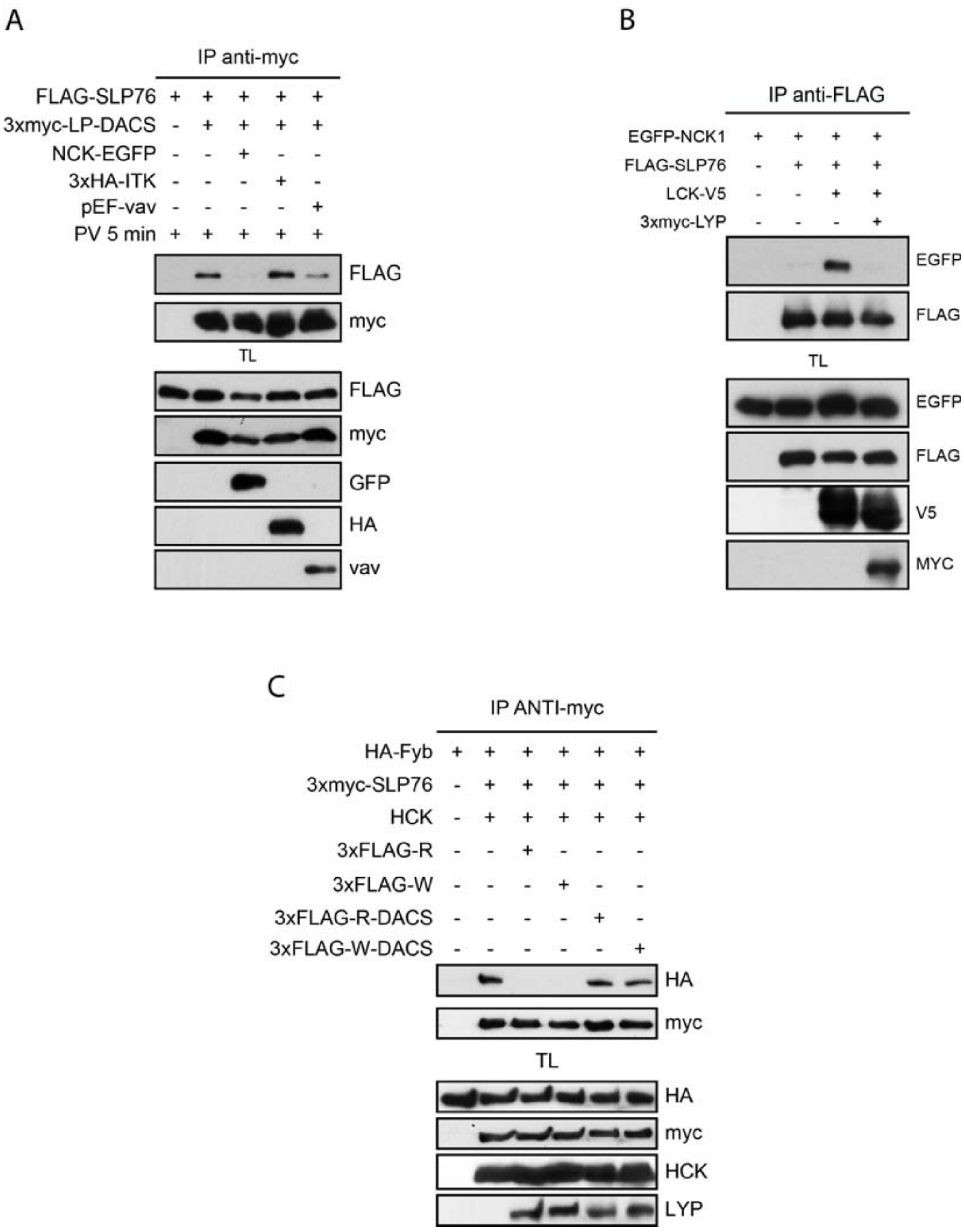

Supplementary Figure 5. LYP impairs the interaction of proteins involved in TCR signaling with SLP76. A, HEK293 cells were transiently transfected with 3xmyc-LP-DACS along with expression plasmids for SLP76, NCK, ITK and vav with different tags, as indicated in the panel. After PV treatment for 5 min, cells were lysed, and LP-DACS was immunoprecipitated with myc Ab bound to sepharose beads. Proteins were separated by SDS-PAGE, and transferred to nitrocellulose membranes. The presence of the different proteins was detected by WB, as indicated. B, As before, FLAG-SLP76 and EGFP-NCK were assayed for binding in the presence of LCK and LYP. C, As in A, interaction of 3xmyc-SLP76 with HA-FYB in cells HEK293 co-transfected with HCK and FLAG-LYP-R620 or FLAG-LYP-W620, as indicated.

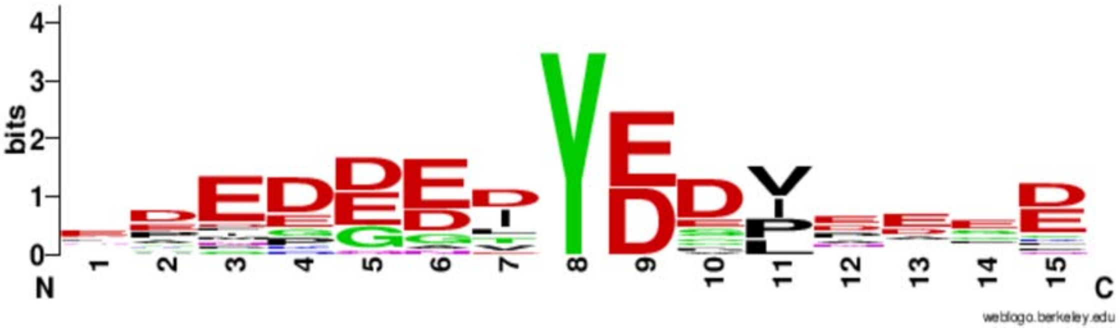

Supplementary Figure 6. Sequence logo of the peptides targeted by LYP in the potential substrates studied. This logo was generated using the WebLogo software (2) with the peptides containing the tyrosines in the following proteins: SKAP2 (Y75, Y237 and Y261), SKAP1 (Y232 and Y271); HS1 (Y378 and Y397), SLP76 (Y113, Y128 and Y145), Vav1 (Y160, Y174), FYB (Y462, Y571, Y595, Y625, Y651 and Y771). The Y axis indicates the degree of conservation of the amino acids, and the X axis the position of the amino acid.

REFERENCES

1. Yu X, Chen M, Zhang S, Yu ZH, Sun JP, Wang L, Liu S, Imasaki T, Takagi Y, Zhang ZY. Substrate specificity of lymphoid-specific tyrosine phosphatase (Lyp) and identification of Src kinase-associated protein of 55 kDa homolog (SKAP-HOM) as a Lyp substrate. *J Biol Chem* (2011) 286:30526–30534. doi: 10.1074/JBC.M111.254722
2. Crooks GE, Hon G, Chandonia J-M, Brenner SE. WebLogo: A Sequence Logo Generator. doi: 10.1101/gr.849004
